# Stable Mott Polaron State Limits the Charge Density in Lead Halide Perovskites

**DOI:** 10.1021/acsenergylett.2c01949

**Published:** 2022-12-08

**Authors:** Heng Zhang, Elke Debroye, Beatriz Vina-Bausa, Donato Valli, Shuai Fu, Wenhao Zheng, Lucia Di Virgilio, Lei Gao, Jarvist M. Frost, Aron Walsh, Johan Hofkens, Hai I. Wang, Mischa Bonn

**Affiliations:** † Max Planck Institute for Polymer Research, Ackermannweg 10, 55128Mainz, Germany; ‡ Department of Chemistry, KU Leuven, Celestijnenlaan 200F, 3001Leuven, Belgium; § Department of Physics, Imperial College London, Exhibition Road, LondonSW7 2AZ, United Kingdom; ∥ School of Physics and Key Laboratory of MEMS of the Ministry of Education, Southeast University, Nanjing211189, China; ⊥ Department of Materials, Imperial College London, Exhibition Road, LondonSW7 2AZ, United Kingdom

## Abstract

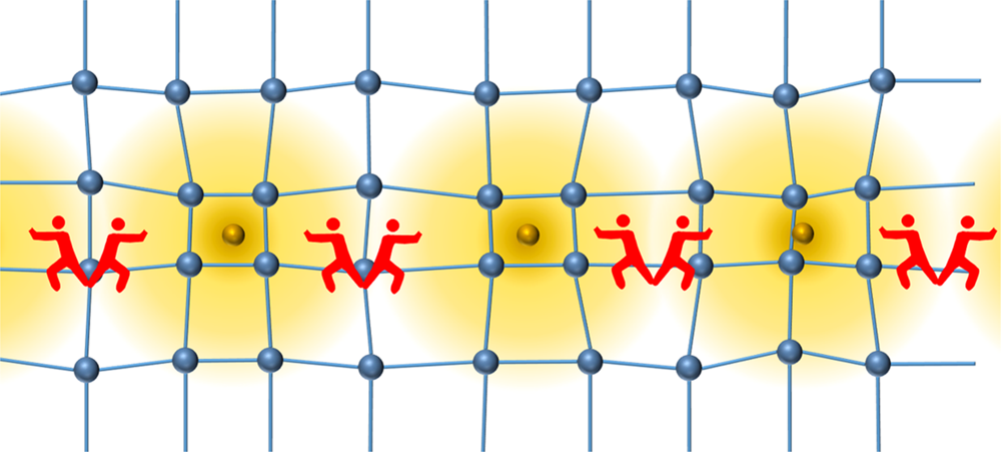

Large polarons are known to form in lead halide perovskites (LHPs). Photoinduced isolated polarons at low densities have been well-researched, but many-body interactions at elevated polaron densities, exceeding the Mott criterion (i.e., Mott polaron density), have remained elusive. Here, employing ultrafast terahertz spectroscopy, we identify a stable Mott polaron state in LHPs at which the polaron wavefunctions start to overlap. The Mott polaron density is determined to be ∼10^18^ cm^–3^, in good agreement with theoretical calculations based on the Feynman polaron model. The electronic phase transition across the Mott density is found to be universal in LHPs and independent of the constituent ions. Exceeding the Mott polaron density, excess photoinjected charge carriers annihilate quickly within tens to hundreds of picoseconds, before reaching the stable and long-lived Mott state. These results have considerable implications for LHP-based devices and for understanding exotic phenomena reported in LHPs.

Low-temperature solution-processed lead halide perovskites (LHPs) have shown superior performance in various electro-optic applications, such as light-emitting diodes (LEDs)^1,2^ and photovoltaic cells.^3,4^ These devices rely on the interconversion of photons and mobile charge carriers. Due to the ionic and soft nature of the perovskite structure, the crystal lattice is readily polarized and distorted around an injected charge carrier. The charge carrier becomes thus dressed by a local lattice deformation, forming a polaron quasiparticle. Because of the weak-to-intermediate electron–phonon interaction in LHPs, large polarons develop, with the lattice distortion spanning over multiple lattice constants, i.e., a few nanometers. Large polaron formation has been proposed in LHPs to underpin their fascinating optoelectronic properties,^5^ including long carrier lifetimes,^6^ good charge mobilities,^7^ and high defect tolerance.^8^ The polarized crystal structure could act as a protective “shield” to effectively screen the Coulombic interactions between the charge carriers and ionized defects, phonons, and among themselves. So far, the spectroscopic and transport studies of polarons in LHPs have mainly focused on the low polaron density regime (∼10^16^ cm^–3^),^9−11^ relevant for, for instance, solar cell applications, where large polarons are, on average, well-separated.

LHPs have also shown great potential in high-excitation optoelectronic applications, including LEDs,^1^ lasing,^12^ and concentrator photovoltaics,^13^ where the photoinjected carrier density commonly exceeds 10^18^ cm^–3^. In this high-density regime, the distorted lattice pattern from different polarons may start to interfere as polarons approach each other. The resultant polaron–polaron interaction significantly impacts the optoelectronic properties, including charge transport and carrier lifetime. For example, strong Auger recombination is typical for LHPs, leading to an efficiency roll-off in, e.g., LEDs.^14,15^ Extremely long-lived hot carriers with lifetime >100 ps have been reported in LHPs^16^ at carrier densities exceeding 10^18^ cm^–3^. Frost et al. attributed this delayed hot-carrier cooling at high fluence to a Mott transition, defined at the carrier density where polaron wavefunctions overlap.^17^ This critical density is also referred to as the Mott polaron density,^17,18^ being analogous to the metal–nonmetal transition that is observed in doped semiconductors.^19^ However, the specific Mott transition and the transport properties in this high-excitation regime have remained largely unexplored experimentally for LHPs, despite their importance and relevance for many applications.

In this work, employing ultrafast terahertz (THz) spectroscopy, we report the observation of an exotic, stable, and long-lived Mott polaron state in LHPs, at which the polaron wavefunctions start to overlap. We quantify the Mott polaron density *N*_Mott_ to be ∼10^18^ cm^–3^, in line with theoretical calculations from a numerical solution to the Feynman polaron model. The electronic phase transition across the Mott density is found to be universal in LHPs and independent of the detailed chemical composition, representing an intrinsic property of LHPs. With increasing excitation density, the excess photoinjected charge carriers beyond *N*_Mott_ annihilate quickly, within tens to hundreds of picoseconds depending on the temperature, to eventually relax to the stable Mott state. These results shed light on the intrinsic polaron many-body effects in LHPs, which impact light-concentrated optoelectronic devices.

LHPs have the general chemical formula of ABX_3_, in which A is an organic or inorganic monovalent cation (e.g., methylammonium MA^+^, formamidinium FA^+^, or Cs^+^), B is a bivalent cation (here, lead), and X is a monovalent halide anion (such as Cl^–^, Br^–^, I^–^). As shown in Figure 1a, the crystal structure of LHPs consists of corner-shared octahedra [BX_6_]^4–^ with the A cations sitting in the inter-octahedral voids. In this work, three LHPs (i.e., MAPbI_3_, γ-CsPbI_3_, and CsPbBr_3_) are prepared as model systems and spin-coated on the fused silica substrates for THz measurements (see Methods for sample preparation). The UV–vis absorption spectra are measured and shown in Figure S1, and the inferred optical properties (e.g., bandgap) are consistent with previous reports. During the THz measurements, the samples are kept under a high vacuum in a cryostat with a background pressure of ∼2 × 10^–4^ mbar. We observe no sample degradation during the THz measurements for all samples. More particularly, the optically active black orthorhombic phase γ-CsPbI_3_ remains intact.

**Figure 1:**
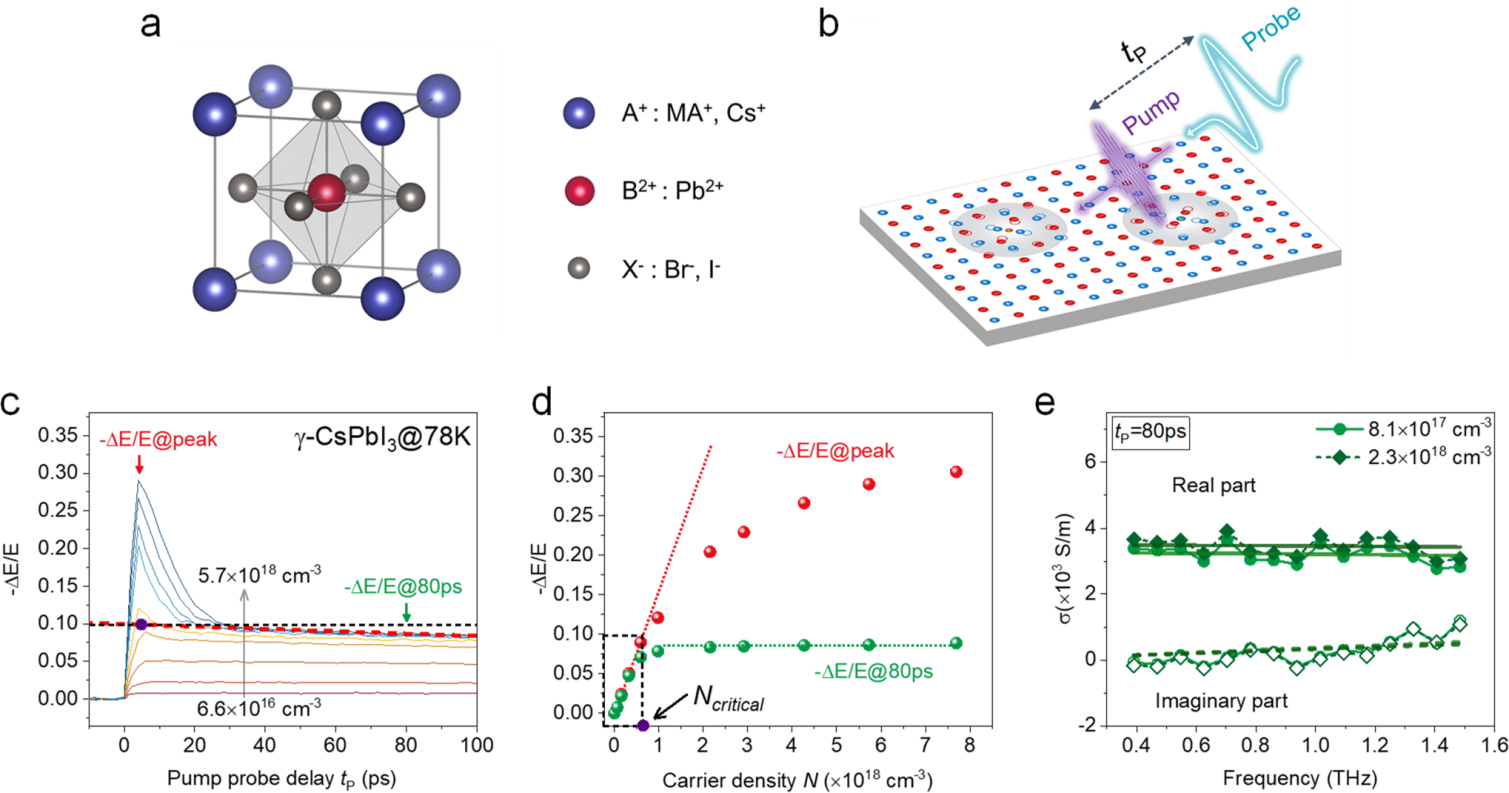
Carrier density saturation in black γ-CsPbI_3_. (a) Atomic unit cell of lead halide perovskites; the three different samples used for this study are γ-CsPbI_3_, MAPbI_3_, and CsPbBr_3_. (b) Schematic illustration of the optical pump–THz probe (OPTP) experiment. (c) Time-dependent −Δ*E*/*E* signals (reflecting carrier densities) at different fluences, in black γ-CsPbI_3_ at 78 K, with a pump photon energy of 3.10 eV. The red dashed line is a linear extrapolation of the slow decay between 40 ps and 100 ps at the highest fluence to zero delay. The black dashed line denotes the corresponding signal at the peak −Δ*E*/*E* (the purple dot). (d) Intensity of −Δ*E*/*E* at the peak and at *t*_P_ = 80 ps in (c) as a function of the initially photoinjected carrier density. The red dotted line is the linear fit to the peak −Δ*E*/*E* values in the low carrier density regime. The green dashed line is the constant fit to −Δ*E*/*E* at *t*_P_ = 80 ps in the high carrier density regime. *N*_critical_ is defined as the carrier density at which the peak −Δ*E*/*E* value is the same as the cross point (the purple dot) in (c). (e) Frequency-resolved complex photoconductivity spectra consisting of real and imaginary components measured at *t*_P_ = 80 ps with initial carrier densities of 8.1 × 10^17^ cm^–3^ and 2.3 × 10^18^ cm^–3^. The solid and dashed lines represent the Drude fits.

To investigate the polaron dynamics in LHPs, we use contact-free optical pump–THz probe (OPTP) spectroscopy and THz time-domain spectroscopy (THz-TDS). A detailed description of both measurements is included in the Methods. Briefly, as shown in Figure 1b, in the OPTP measurements, the perovskite sample is first excited by an ultrashort optical (“pump”) pulse with 50 fs pulse duration to photoinject charge carriers into the conduction band (i.e., electrons) and valence band (i.e., holes). After a controlled delay time *t*_P_, a single-cycle THz pulse as the “probe” with ∼1 ps duration propagates collinearly through the excited sample and interacts with the photoinjected charge carriers. This interaction causes an attenuation of the THz field. The attenuation provides a direct measure of the conductivity σ of the sample, which in turn is defined by the product of the elementary charge *e*, the carrier density *N*, and the carrier mobility μ: σ = *eN*_e_μ_e_ + *eN*_h_μ_h_ (with *N*_e_ = *N*_h_, where the subscripts “e” and “h” stand for electrons and holes, respectively). As such, THz spectroscopy provides direct access to the charge density following optical excitations, with high time resolution (∼200 fs).^20,21^ The transmitted THz electric field following the excitation pulse *E*_exc_(*t*) is detected coherently by another 800 nm sampling pulse (pulse duration 50 fs) in a ZnTe crystal via the electro-optic effect. The transmitted field in the absence of the excitation pulse *E*_0_(*t*) is also determined. It can be demonstrated that the quasi-instantaneous sample conductivity at time *t*_P_ can be approximated by σ(*t*_P_) ∝ −Δ*E*(*t*_P_)/*E*_0_, with Δ*E*(*t*_P_) = *E*_exc_(*t*_P_) – *E*_0_.^22^ In addition, by mapping out the change of the full THz waveform at a fixed pump-sampling delay time *t*_P_ and then performing the Fourier transformation, the complex frequency-resolved photoconductivity spectrum σ(ω) at *t*_P_ can be obtained.

In Figure 1c, we show the fluence-dependent OPTP dynamics of black γ-CsPbI_3_ at 78 K with varying photoinjected carrier density *N* over nearly 2 orders of magnitude (from 6.6 × 10^16^ cm^–3^ to 5.7 × 10^18^ cm^–3^). *N* is inferred from the incident photon density *N*_photon_, the sample absorption *A*, and the photon-to-charge quantum yield Φ: *N* = Φ*N*_photon_*A*. Φ is around 30% for black γ-CsPbI_3_.^10^ An independent determination of the quantum yield from the plasma frequency obtained from the THz conductivity spectra confirms this value (see Supporting Information). Due to their similar effective masses,^23^ both electrons and holes are included here, as they contribute almost equally to the measured photoconductivity. With increasing the carrier density *N*, distinct features appear in the OPTP dynamics: in the low excitation regime (*N* < ∼7 × 10^17^ cm^–3^), the amplitude of the photoconductivity increases linearly with excitation density and shows little decay within our time window of ∼1 ns (see the inferred lifetime at low fluences in Figure S3 in the Supporting Information). The observed low charge carrier recombination rate is in line with our previous report on the carrier dynamics of γ-CsPbI_3_,^10^ and is also consistent with the picture of large polaron formation, which reportedly screens the charge carriers from defects and other charge carriers.^6,10,20,24^ When the polaron density is low, the overlap between their wavefunctions is small, thus limiting the bimolecular recombination rate.

In the high excitation regime (with *N* > ∼7 × 10^17^ cm^–3^), a rapid decay appears with a time constant spanning from several to tens of ps, then followed by a long-lived signal again as in the low excitation regime (see Figure S3 in the Supporting Information for fluence-dependent OPTP dynamics at long time scales). The peak photoconductivity increases continuously with photoinjected carrier density, yet the signals quickly decay to the same level, independent of the initial excitation density. This unique carrier-density-dependent photoconductivity evolution is summarized in Figure 1d by plotting the signal amplitude at two representative time cuts: at the photoconductivity peak, and at 80 ps after photoexcitation. This figure reveals the existence of a critical charge carrier density *N*_critical_: below *N*_critical_, the photoconductivity increases linearly with *N*, and it is long-lived; exceeding *N*_critical_, the peak photoconductivity is still increasing with *N*, yet the conductivity at later times (e.g., *t*_P_ = 80 ps) reaches a plateau. To accurately estimate the critical density *N*_critical_, we extrapolate the long-lived signal at the highest fluence (from 40 ps to 100 ps here for γ-CsPbI_3_) back to the peak −Δ*E*/*E* value, as shown in Figure 1c, and then project this value to the corresponding carrier density *N*, i.e., critical density *N*_critical_, by using the linear relation between the peak value of −Δ*E*/*E* and *N* at low fluences. For black γ-CsPbI_3_ investigated here, we find *N*_critical_ = (6.4 ± 1.5) × 10^17^ cm^–3^.

To verify if the long-time photoconductivity for all excitation fluences reaches the same state (with the same density and mobility of charge carriers), we measured full photoconductivity spectra at *t*_P_ = 80 ps. Figure 1e shows two typical complex photoconductivity spectra under two different excitation densities (with *N* = 8.1 × 10^17^ cm^–3^ and 2.3 × 10^18^ cm^–3^). The complex spectra share nearly the same dispersion and intensity (see more data and discussion below). This result indicates that, independent of initial excitation density, for *t*_P_ > 30 ps, the charge carriers reach the same state with the same charge mobility and carrier density, independent of the initial excitation density. We tentatively attribute the fast carrier loss during the first 30 ps in the high excitation regime to Auger recombination, as detailed in the Supporting Information, but cannot exclude other contributions to the decay of the signal.

At the same time, THz-TDS provides an independent way to estimate the critical density, that is, to fit the photoconductivity spectrum by theoretical models (e.g., Drude model or Drude–Smith model) and extract the carrier density (see the spectra and fitting for all three samples in the Supporting Information). For black γ-CsPbI_3_, we fit the photoconductivity spectrum (measured at *t*_P_ = 80 ps at an excitation fluence of 9.35 × 10^17^ cm^–3^) by the Drude model. After correcting for the small density decay occurring up to 80 ps, we find *N*_critical_ = (7.2 ± 1.4) × 10^17^ cm^–3^, in good agreement with the value obtained from the OPTP result. Importantly, we find the critical density is independent of the excitation photon energy (see a comparison of 400 nm and 750 nm excitation in the Supporting Information). This result further confirms the intrinsic origin of the observed critical density.

To test whether the observed carrier density saturation is unique to the black γ-CsPbI_3_, we conducted measurements on the other two typical LHPs, i.e., MAPbI_3_ and CsPbBr_3_, with either the A-site cation or the X-site anion changed compared to γ-CsPbI_3_. Although some details about OPTP dynamics vary for different perovskites (e.g., the carrier lifetime and signal amplitude), the same carrier density saturation phenomenon is observed in MAPbI_3_ and CsPbBr_3_ when the carrier density exceeds a threshold *N*_critical_, as shown in Figure 2a,b. Based on this observation, we can conclude that this effect seems universal in LHPs, including organic–inorganic hybrids and all-inorganic LHPs, independent of the ionic composition. By the same approach utilized in black γ-CsPbI_3_ to extract the critical density from OPTP measurement, the insets of Figure 2a,b show that the obtained *N*_critical_ for MAPbI_3_ and CsPbBr_3_ are (5.7 ± 2.2) × 10^17^ cm^–3^ and (4.1 ± 2.0) × 10^17^ cm^–3^, respectively. By THz-TDS, we extract the *N*_critical_ for MAPbI_3_ and CsPbBr_3_ as (5.8 ± 0.3) × 10^17^ cm^–3^ and (4.7 ± 0.2) × 10^17^ cm^–3^, respectively, taking the density decay into consideration as well (see Supporting Information).

**Figure 2:**
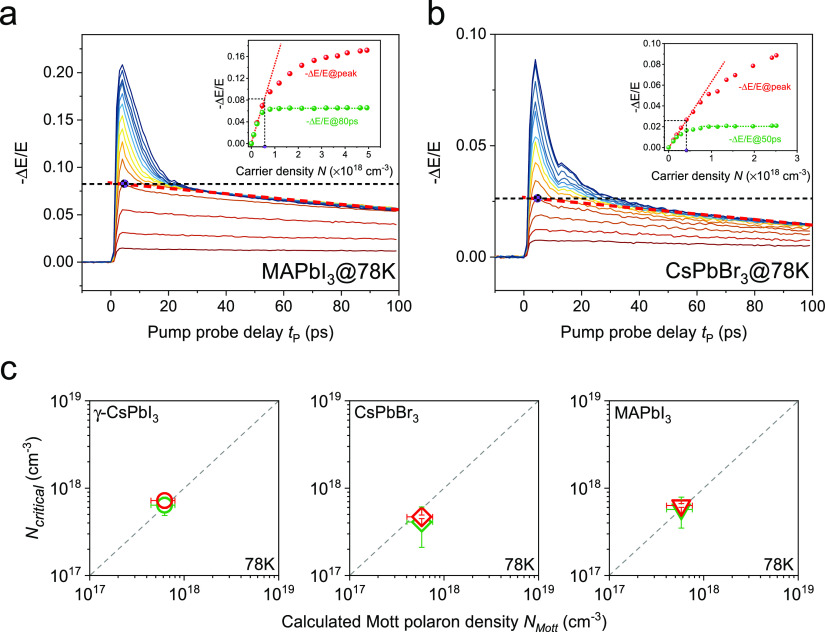
THz measurements in MAPbI_3_ and CsPbBr_3_. (a, b) Fluence-dependent OPTP dynamics in MAPbI_3_ and CsPbBr_3_ at 78 K, with an excitation photon energy of 3.10 eV. Insets: the intensity of −Δ*E*/*E* at the peak and at later time delays (*t*_P_ = 80 ps for MAPbI_3_ and *t*_P_ = 50 ps for CsPbBr_3_) as a function of photoinjected carrier density. (c) Comparison between the experimentally extracted critical density *N*_critical_ and the calculated Mott polaron density based on the Feynman polaron model for γ-CsPbI_3_, MAPbI_3_, and CsPbBr_3_. The green symbols represent *N*_critical_ obtained from the OPTP measurements, while the red symbols are *N*_critical_ extracted from the THz-TDS measurements. The dashed lines indicate *N*_critical_ = *N*_Mott_.

Converting the critical density to the average volume per charge carrier and further the distance *d* between neighboring carriers, we estimate *d* to be around 10 nm. This value is similar to the polaron diameter in LHPs.^17,25^ This simple estimate indicates that *N*_critical_ can be interpreted as the Mott polaron density *N*_Mott_ in LHPs in which neighboring polarons overlap, and it sets the upper limit of the charge carrier density that LHPs can support in thermal equilibrium. To examine this hypothesis, in Figure 2c we correlate the experimentally extracted critical densities *N*_critical_ (from both OPTP and THz-TDS) to the calculated *N*_Mott_ for all three perovskites at 78 K. The Mott polaron density is calculated based on a finite temperature numeric solution to the Feynman polaron model.^26^ The polaron radius is defined following Schultz as the width of the Gaussian polaron wavefunction ansatz in the Feynman model.^27^ Assuming, for simplicity, that the polaron occupies a cube with sides twice the polaron radius, the Mott polaron density is thus inferred. The parameters used to calculate *N*_Mott_ for the three perovskites are shown in Table S3 in the Supporting Information. As can be seen in Figure 2c, a direct correlation between the extracted critical density *N*_critical_ and the Mott polaron density *N*_Mott_ is evident for all three LHPs.

To further test the one-to-one correlation between *N*_critical_ and *N*_Mott_, we perform temperature (*T*)-dependent photoconductivity studies. Based on the Feynman theory, the polaron radius, and thereby *N*_Mott_, is strongly temperature-dependent. In the finite temperature formulation of Feynman’s polaron model, the maximum extent of the polaron is at zero temperature. At increasing temperature, the finite phonon population (and associated polarization fields) further localizes the charge carrier. This relationship, and the accuracy of the Feynman method, was confirmed in a recent quantum Monte Carlo study.^28^ In other words, the polaron size increases as *T* decreases,^17^ as shown schematically in Figure 3a. Correspondingly, the model predicts that the Mott polaron density will increase with *T*. Figure 3b shows the calculated *N*_Mott_ at different *T* for black γ-CsPbI_3_. The calculated Mott polaron density increases substantially, by a factor of ∼4, from 50 K to 300 K. To check the effect of *T* on the OPTP dynamics and critical density, Figure 3c shows the fluence-dependent OPTP measurement in black γ-CsPbI_3_ at 286 K as an example (see more *T*-dependent measurements in the Supporting Information), and the inset displays the extraction of the critical density. With increasing initial excitation density, the signal saturation takes place in hundreds of ps at 286 K, instead of tens of ps at 78 K. The extracted critical density here is (1.47 ± 0.3) × 10^18^ cm^–3^, which is very close to the calculated Mott density of 1.67 × 10^18^ cm^–3^ at 286 K. In Figure 3d, we further show the inferred *N*_critical_ at *T* varying from 78 K to 286 K, which correlates well with *N*_Mott_ obtained from calculations. Note that there is no phase transition^29^ for black γ-CsPbI_3_ below room *T*, and we observe nearly no change in the photon-to-carrier conversion efficiency within this *T* range (see Supporting Information). This *T*-dependent result further supports the existence of a Mott polaron transition in LHPs. This delayed signal saturation at high *T* indicates a slower stabilization at the Mott polaron density, which may be due to increased thermal disorder (anharmonic phonon scattering) at elevated temperatures. The higher Mott density impedes thermal dissipation from hot polarons.^17^

**Figure 3:**
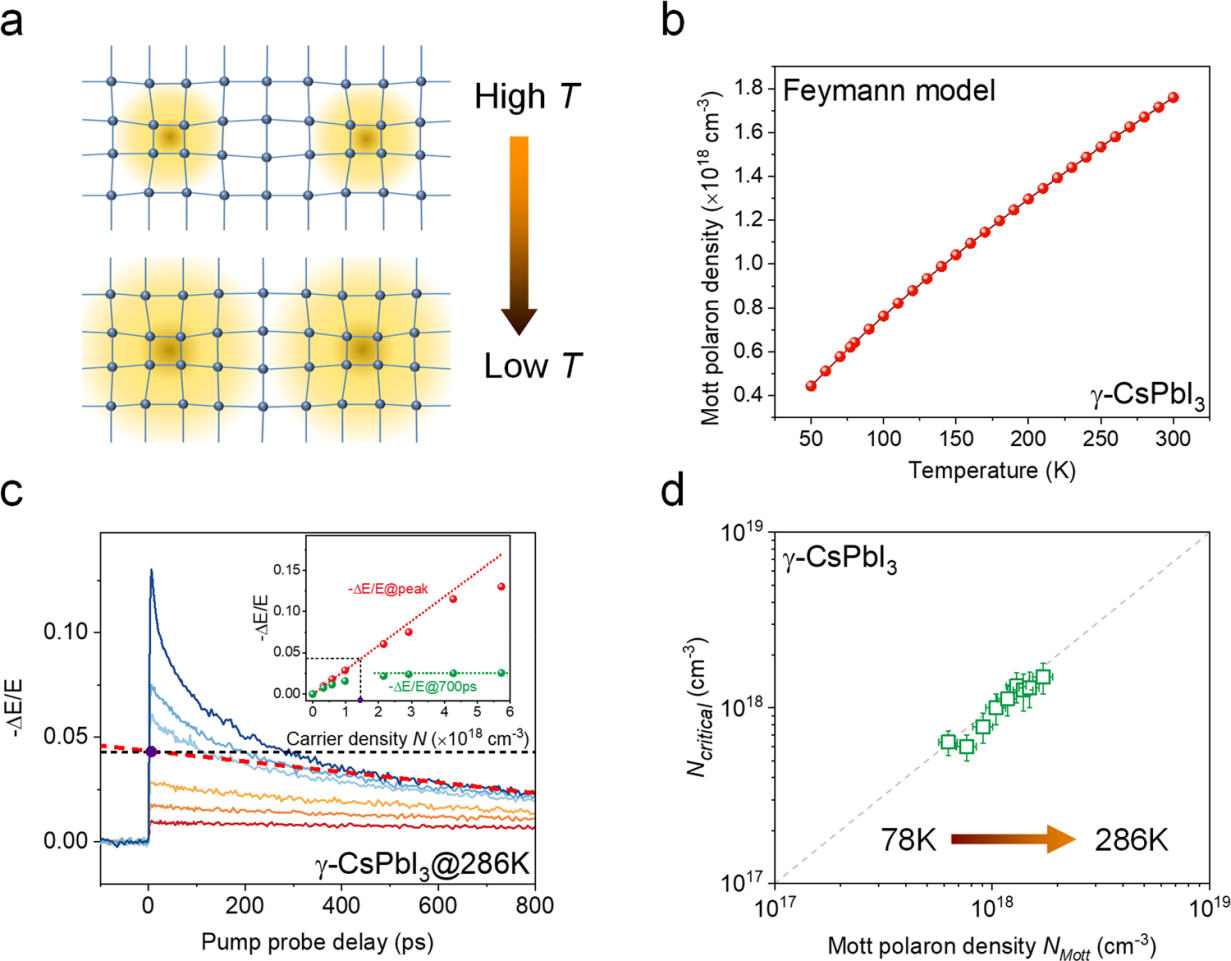
Temperature-dependent critical density in LHPs. (a) Schematic illustration of polaron size evolution upon lowering the temperature. (b) Calculated Mott polaron density as a function of temperature based on the finite temperature Feynman polaron model for black γ-CsPbI_3_. (c) Fluence-dependent OPTP dynamics at 286 K in black γ-CsPbI_3_ with an excitation photon energy of 3.10 eV. Insets: the intensity of −Δ*E*/*E* at the peak and at *t*_P_ = 700 ps as a function of photoinjected carrier density. The dashed red line shows the extrapolation back to zero pump–probe delay from data between 500 ps and 800 ps at high excitation density, as in Figure 1c. (d) Comparison between the experimentally (OPTP) extracted critical density *N*_critical_ and the calculated Mott polaron density at different temperatures for γ-CsPbI_3_. The dashed line indicates where *N*_critical_ and *N*_Mott_ are the same.

After establishing the Mott polaron state and quantifying its density, we characterize the charge transport properties below and above it. In Figure 4a,b, we show two typical photoconductivity spectra of the black γ-CsPbI_3_ at 78 K following two excitation fluences, below and above the Mott density: 1 × 10^17^ cm^–3^ and 2.33 × 10^18^ cm^–3^. Both measurements are conducted at 80 ps following optical excitations to ensure that Mott polaron states are established following exceeding-Mott-density excitations. As we can see, both photoconductivity spectra share the same feature, with a positive, decreasing real part and a positive, increasing imaginary part with frequency. This is a clear signature of delocalized band transport and can be well fitted by the Drude model. The solid and dashed lines in Figure 4a,b show the real and imaginary conductivity, respectively, and their description using the Drude model. The Drude model has two free parameters: carrier density *N* and carrier scattering time τ. Figure 4c summarizes the excitation-density-dependent *N* and τ. The obtained carrier density grows rapidly and then saturates at ∼6.4 × 10^17^ cm^–3^, consistent with the inferred Mott density above in γ-CsPbI_3_ at 78 K. Furthermore, the extracted scattering time shows a strong fluence-dependent transition, from an almost fluence-independent high scattering value of over 40 fs at low polaron density (for absorbed photon density below ∼40% of Mott density) to less than 20 fs above the Mott density. This result indicates that even at the Mott polaron density, delocalized band transport still prevails in LHPs, but that some additional scattering pathway has been activated. Most likely, this is carrier–carrier scattering now that the polaron wave functions are overlapping. The enhanced carrier scattering interaction reduces the charge mobility (μ = *e*τ/m*, with m* as the effective mass) from ∼570 cm^2^ V^–1^ s^–1^ below to ∼350 cm^2^ V^–1^ s^–1^ above the Mott density, despite a maximum ∼25% reduction in effective mass above the Mott density (see Supporting Information).

**Figure 4:**
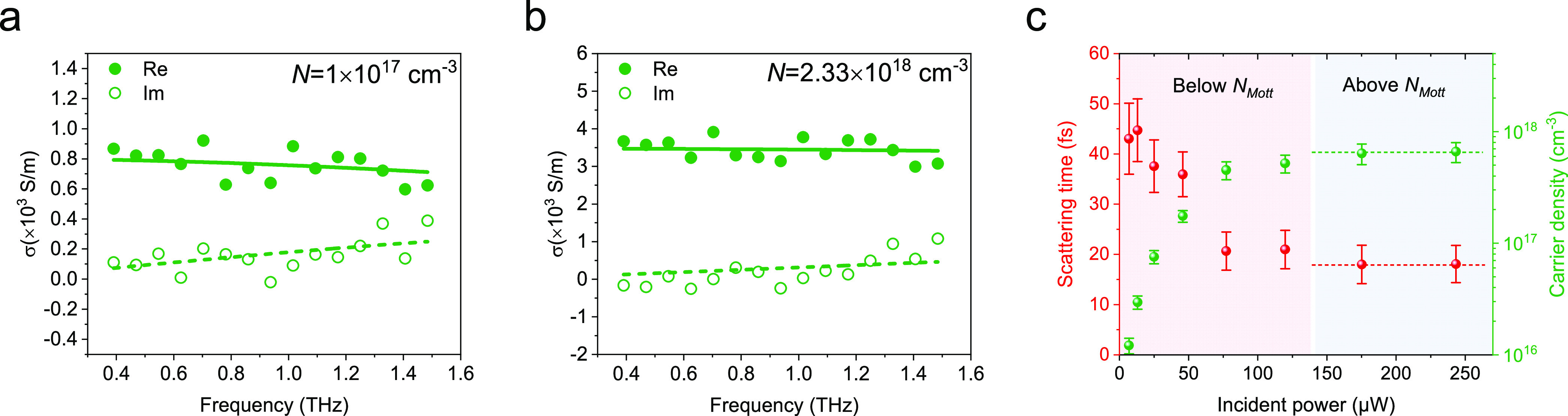
Fluence-dependent complex photoconductivity for black γ-CsPbI_3_ measured at *t*_P_ = 80 ps at a temperature of 78 K. (a, b) Frequency-resolved photoconductivity spectra (filled and empty circles) and Drude model fits (solid and dashed lines) at initial photogenerated carrier densities of 1 × 10^17^ cm^–3^ and 2.33 × 10^18^ cm^–3^. (c) Extracted scattering times and carrier densities from the Drude fits at different incident fluences.

The observation of the Mott polaron state sets the upper limit on the available large polaron population (i.e., *N*_Mott_) that LHPs can host. Theoretically, Emin predicted an emerging repulsive interaction acting as an energy barrier between the oppositely charged large polarons when the polaron separation is small enough.^30^ Such repulsive polaronic interactions are balanced by the electron–hole Coulomb attraction, determining the spatial energy landscape of polaron states. These balanced interactions underlie the formation of the stable Mott polaron state in LHPs. We note that the “ferroelectric” nature of the polarons proposed by Zhu et al. may further impact the electrostatic stabilization of the Mott polaron state.^31^ Besides the low trapping rate (so-called “defect tolerance” effect) reported in LHPs linked to the dielectric screening that also drives large polaron formation, our discussion here can also explain the weak bimolecular recombination rate (i.e., free electron–hole recombination) and thus long carrier lifetime in LHPs in solar cell applications.

At high excitation densities (*N* > 10^18^ cm^–3^), unexpectedly strong Auger recombination in LHPs is often deduced from spectroscopic studies, including THz spectroscopy^10,32^ and transient absorption spectroscopy.^33,34^ The inferred Auger coefficient is almost 2 orders of magnitude higher in LHPs than in conventional semiconductors with similar bandgaps.^14^ This strong Auger recombination leads to the efficiency roll-off in LEDs^15^ and concentrator photovoltaics^13^ when the carrier density is above ∼10^18^ cm^–3^. In addition, an exceptionally accelerated dynamics decay is observed at lower temperatures.^32,35^ Yet, the underlying mechanism for the strong Auger recombination in LHPs is still under debate. These phenomena can be rationalized by the formation of the stable Mott polaron state observed in this work. For densities exceeding *N*_Mott_, the enforced overlap of polarons weakens the protection of the induced charges.^36^ This results in an enhanced electron–hole interaction and, therefore, a faster population annihilation rate.^11^ At lower temperatures, the population reduces to the Mott polaron density faster, within tens of ps, in line with the enhanced Auger recombination at low *T*.

The observed Mott density may also be closely related to the carrier densities at which long hot carrier lifetimes were reported in LHPs. At a carrier density of 6 × 10^18^ cm^–3^, Yang et al. reported hot carrier lifetimes up to 100 ps in MAPbI_3_ and FAPbI_3_.^16^ Frost et al. proposed that this slowed hot carrier cooling at high fluence originates from the polaron overlap and shared phonon subpopulation.^17^ Our results corroborate this proposal. Due to the destabilized polaron protection above the Mott polaron density, the hot carrier–longitudinal optical (LO) phonon interaction increases with fluence. However, polaron overlap at the stable Mott state impedes the efficient phonon diffusion away from the hot carriers, which, in turn, reheats the relaxed carrier population. As a consequence, the hot carriers in LHPs can be maintained for a long time.

In summary, we observe the formation of a stable Mott polaron density in lead halide perovskites in the high-excitation regime (>10^18^ cm^–3^). This phenomenon is universal and independent of the constituents’ ionic nature. Quantitative agreement is found for the Mott polaron density between the experiments and the calculations from the temperature-dependent Feynman variational polaron theory. Above the Mott polaron density, the photoinjected excess carriers annihilate within tens to hundreds of ps depending on the temperature. Our results are crucial for understanding the intrinsic optoelectronic properties of LHPs and shed light on the performance of light-concentrated optoelectronic devices.

## Methods

### Synthesis of Lead Halide Perovskites

All reagents were used as received without any further purification. Methylammonium iodide >99.99% (Greatcell Solar Materials); lead(II) iodide ultra-dry 99.999% (metals basis) (Thermo Scientific); *N,N*-dimethylformamide (DMF) 99.8% extra dry over AcroSeal molecular sieves (Acros Organics); cesium bromide 99.9% metals basis (Thermo Scientific); lead(II) bromide ≥98% (Sigma-Aldrich); dimethyl sulfoxide (DMSO) 99.7+% extra dry over AcroSeal molecular sieves (Acros Organics); chlorobenzene 99+% for spectroscopy (Acros Organics); γ-butyrolactone (GBL) ReagentPlus ≥99% (Sigma-Aldrich); and cesium iodide 99.9% trace metals basis (Sigma-Aldrich).

The substrates for the film deposition consisted of fused silica substrates (1 cm × 1 cm) which were washed with isopropanol, dried, and left in an ozone reactor chamber for 30 min.

Thin films of CH_3_NH_3_PbI_3_ were prepared by a modified protocol according to a previous report.^1^ CH_3_NH_3_I (0.0795 g) and PbI_2_ (0.2301 g) were mixed in a 1:1 stoichiometric ratio in anhydrous DMF (1 mL) to give a 0.5 M solution, which after stirring at room temperature for at least 20 min produced a clear CH_3_NH_3_PbI_3_ solution. The solution was filtered with a 0.20 μm pores hydrophilic PTFE filter. The perovskite film deposition was performed under an inert atmosphere inside a glovebox. The CH_3_NH_3_PbI_3_ solution (50 μL) was deposited on the fused silica glass and then spin-coated at 5000 rpm for 30 s. The solvent was evaporated at 100 °C for 10 min (still in the glovebox), resulting in a clear brown film. The more diluted CH_3_NH_3_PbI_3_ film was obtained by diluting 15 μL of the former CH_3_NH_3_PbI_3_ solution in 45 μL of anhydrous DMF, keeping the rest of the protocol the same. The film was stored in the dark under an inert atmosphere until further measurements.

CsPbBr_3_ thin films were prepared by spin-coating a 0.5 M DMSO solution of CsBr and PbBr_2_ (1:1 stoichiometric ratio). The solution was prepared by mixing CsBr (0.1064 g) and PbBr_2_ (0.1835 g) in 1–1.5 mL of dry DMSO at around 70 °C. The solution was then filtered with a 0.20 μL pore PTFE hydrophilic filter. For obtaining the thin film, 70 μL of the perovskite solution was spin-coated on the fused silica glass, in a glovebox. Subsequent spin-coating at 1000 rpm for 10 s, followed by 60 s at 3000 rpm, with the addition of 125 μL of chlorobenzene 35 s before the end of the program, resulted in the desired thin film after solvent evaporation (for 10 min on a hot plate at 60 °C). The diluted CsPbBr_3_ thin film was prepared by diluting the former perovskite solution by 25% in volume, while retaining all other steps. Both of the films were annealed outside the glovebox for 30 min at 150 °C. The films were stored in the dark under an inert atmosphere until further measurements.

CsPbI_3_ thin films were prepared from a 1:1 stoichiometric solution of CsI and PbI_2_. The two salts were mixed in a 4:1 DMSO:GBL solution (1 M) at 60 °C overnight and then filtered with a 0.20 μL pore PTFE hydrophilic filter. 70 μL of the clear solution was spin-coated on the substrate using the following program: 300 rpm for 30 s, 1000 rpm for 20 s, and finally 4000 rpm for 60 s. The coated substrate was heated first at 160 °C for 1 h and then at 320 °C for 15 min, until the substrate changed color to the desired black phase. The whole procedure was carried out under an inert atmosphere (N_2_) in a glovebox. The samples were stored in the dark under an inert atmosphere until for further measurements.

### Optical Pump–THz Probe (OPTP) Spectroscopy

The OPTP setup is driven by a commercial, regenerative amplified, mode-locked Ti:sapphire femtosecond laser with 1 kHz repetition rate. In the OPTP measurement, as shown in Figure 1b, the perovskite sample is first pumped by a 400 nm (∼3.10 eV) optical pulse with a 50 fs pulse duration. The pump pulse is generated from an 800 nm (∼1.55 eV) pulse by second-harmonic generation in a beta barium borate (BBO) crystal. After a pump–probe delay time *t*_P_, the THz pulse propagates collinearly with the pump pulse and transmits through the sample. The single-cycle THz pulse is generated from an 800 nm pulse by optical rectification in a 1-mm-thick ZnTe crystal. The transmitted THz electric field waveform *E*(*t*) is detected coherently in a second ZnTe crystal by another 800 nm sampling pulse via the electro-optic effect. The photoconductivity dynamics σ(*t*_P_) of the sample is thus obtained by reading out the pump-induced relative change of THz peak electric field −Δ*E*/*E*_0_(*t*_P_) as a function of *t*_P_, based on the thin-film approximation:^37^
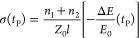
Here, Δ*E* = *E*_exc_ – *E*_0_, in which *E*_0_ and *E*_exc_ are the transmitted THz electric fields before and after the pump. *n*_1_ and *n*_2_ are the refractive indices in front of and behind the sample. *Z*_0_ is the free space impedance. *l* is the thin-film thickness (∼200 nm for all films in this study).

### THz Time-Domain Spectroscopy (THz-TDS)

THz-TDS is conducted to obtain the frequency-resolved photoconductivity spectra. Instead of only recording the change of the THz peak electric field after the photoexcitation, one can detect the whole THz waveform with (*E*_pump_(*t*)) and without (*E*_0_(*t*)) optical excitations (and thus their difference Δ*E*(*t*) = *E*_pump_(*t*) – *E*_0_(*t*)) at a fixed pump–sampling delay time by moving the optical delay lines for the pump beam and sampling beam simultaneously.^38^ By Fourier transformation of *E*_0_(*t*) and Δ*E*(*t*), the complex photoconductivity spectra σ(ω) are achieved as follows:
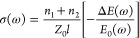


## Data Availability

All data needed to evaluate the conclusions in the paper are present in the paper and/or the Supporting Information.

## Supporting Information Available

UV–vis absorption, determination of photon-to-carrier quantum yield, lifetime estimation at low fluence, discussion on the fast decay of OPTP dynamics, extraction of critical density by THz-TDS, extracted critical densities at different pump wavelengths and different temperatures, Mott polaron density calculations, photon-to-carrier conversion ratio at different temperatures, and density-dependent effective mass and charge mobility in γ-CsPbI_3_ (PDF)
