# Supplementary material for: Stable Mott Polaron State Limits the Charge Density in Lead Halide Perovskites
Source: ACS Energy Lett. 2022 Dec 8;8(1):420–8. doi: 10.1021/acsenergylett.2c01949 (PMC9841606; doi:10.1021/acsenergylett.2c01949)
Supplement: Supplementary file 1 — nz2c01949_si_001.pdf [file nz2c01949_si_001.pdf]

Stable Mott Polaron State Limits the Charge Density in Lead Halide Perovskites

Heng Zhang,[1] Elke Debroye,[2] Beatriz Vina-Bausa,[3] Donato Valli,[2] Shuai Fu,[1] Wenhao Zheng,[1] Lucia Di Virgilio,[1] Lei Gao,[1,4] Jarvist M. Frost,[3] Aron Walsh,[5] Johan Hofkens,[2] Hai I. Wang,[1*] Mischa Bonn[1*]

[1] Max Planck Institute for Polymer Research, Ackermannweg 10, 55128 Mainz, Germany

[2] Department of Chemistry, KU Leuven, Celestijnenlaan 200F, 3001 Leuven, Belgium

[3] Department of Physics, Imperial College London, Exhibition Road, London SW7 2AZ, United Kingdom

[4] School of Physics and Key Laboratory of MEMS of the Ministry of Education, Southeast University, Nanjing 211189, China

[5] Department of Materials, Imperial College London, Exhibition Road, London SW7 2AZ, United Kingdom

Corresponding to: wanghai@mpip-mainz.mpg.de, bonn@mpip-mainz.mpg.de

Section 1: The UV-vis absorption spectrum and bandgap estimation

Section 2: Determination of photon-to-carrier quantum yield Φ

Section 3: Lifetime estimation at low fluence for black γ-$CsPbI_3$

Section 4: Discussion on the fast decay of OPTP dynamics

Section 5: The extraction of critical density by THz-TDS

Section 6: Extracted critical densities at different pump wavelength

Section 7: Mott polaron density calculation based on Feynman polaron model

Section 8: The extraction of critical density at different temperatures

Section 9: The photon-to-carrier conversion ratio at different temperature

Section 10: Density-dependent effective mass and charge mobility in γ-$CsPbI_3$

Section 1: The UV-vis absorption spectrum and bandgap estimation

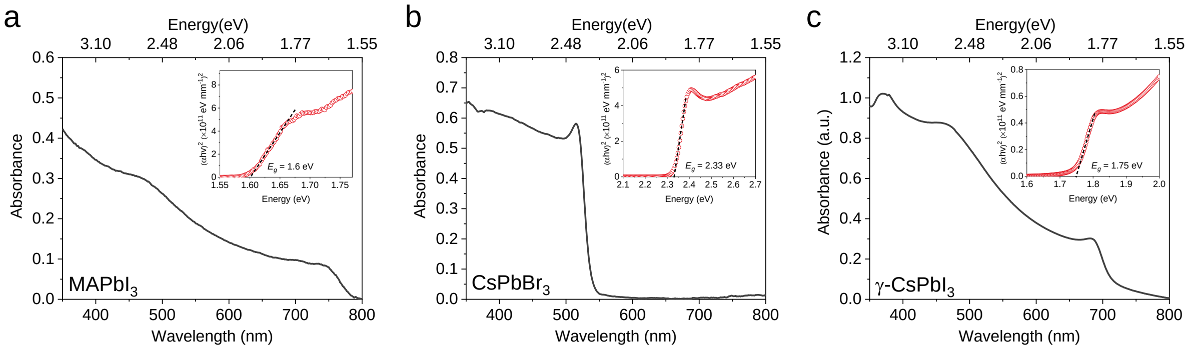

Fig. S1. The UV-vis absorption spectra for $MAPbI_3$, $CsPbBr_3$ and black γ-$CsPbI_3$ measured at room temperature. The insets are the Tauc plots for estimating the bandgaps. The absorption spectra and the inferred bandgaps are consistent with previous reports[1-3].

Section 2: Determination of photon-to-carrier quantum yield Φ

To assess the photon-to-carrier quantum yield, we conducted the THz-TDS to obtain the photoconductivity spectrum and then fitted it with the Drude or Drude-Smith model, commonly used to characterize the charge transport in solids[4]. The Drude model describes the frequency-dependent response of charge carriers undergoing completely independent, momentum-randomizing scattering events. The formula reads:

$$\sigma(\omega) = \frac{\omega_p^2 \varepsilon_0 \tau_s}{1 - i\omega\tau_s} \tag{1}$$

where ω_p is the plasma frequency, which is directly related to the carrier density N by $\omega_p = \sqrt{\frac{N}{\varepsilon_0 \cdot m^*}}e$ (m^* is the effective mass), ε_0 the vacuum permittivity, τ_s the scattering time and ω the angular frequency.

For charge carriers subjected to spatial confinement e.g. from grain boundaries or interfaces, they may experience backscattering effect, which can be well decribed by a modified Drude model: the Drude-Smith model. The model follows:

$$\sigma(\omega) = \frac{\omega_p^2 \varepsilon_0 \tau_s}{1 - i\omega\tau}\left(1 + \frac{c}{1 - i\omega\tau}\right) \tag{2}$$

Here, parameter c ($-1 < c < 0$) is introduced to account for the different extent of backscattering. For $c = -1$, the charge carriers are going through 100% backscattering, while $c = 0$, the Drude-Smith model is simplified to the Drude model.

By fitting these models to the photoconductivity spectra, we can extract the plasma frequency and then the carrier density N. The photon-to-carrier quantum yield Φ can be defined as: $\Phi = \frac{N}{N_{abs}}$, with N_{abs} as the absosrbed photon density.

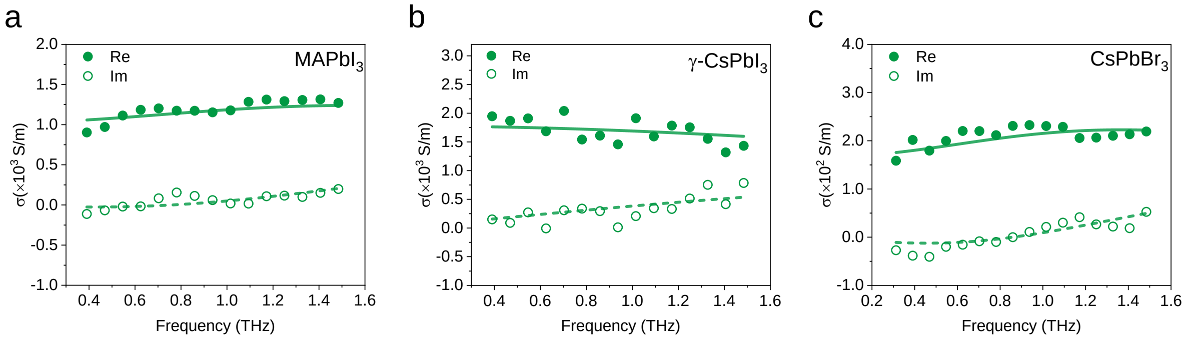

Fig. S2. The frequency-resolved photoconductivity spectra for $MAPbI_3$, $CsPbBr_3$ and black γ-$CsPbI_3$ measured at 78 K.

Table S1. The estimation of photon-to-carrier quantum yield Φ for three perovskites

Sample	$N_{Absorbed}$ (cm^{-3})	N_{Fitted} (cm^{-3})	Φ (%)
$MAPbI_3$	8.30×10^{17}	1.27×10^{17}	~15
γ-$CsPbI_3$	5.87×10^{17}	1.75×10^{17}	~30
$CsPbBr_3$	6.30×10^{17}	0.51×10^{17}	~8

Here, $N_{Absorbed}$ is the absorbed photon density by taking into account the incident photon density and sample absorption, N_{Fitted} is the extracted carrier density from the fitting to the photoconductivity spectra.

Section 3: Lifetime estimation at low fluence for black γ-$CsPbI_3$

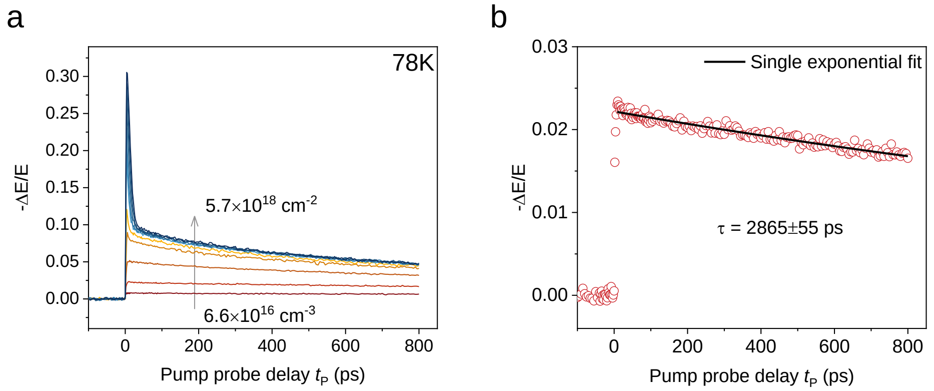

Fig. S3. a, The fluence-dependent OPTP dynamics for black γ-$CsPbI_3$ at 78 K. **b,** The OPTP dynamics at carrier density of 1.58×10^{17} cm^{-3}. The black solid line is the single exponential fit with the inferred lifetime of around

2.9 ns.

Section 4: Discussion on the fast decay of OPTP dynamics

Here we take $MAPbI_3$ as an example to discuss the nature of the fast decay in the fluence-dependent OPTP dynamics. We attribute the fast decay to the combination of charge carrier population decay (following Auger recombination) and the reduction of the charge mobility at high fluences due to the enhanced carrier-carrier or carrier-phonon interactions[5,6]. On top of that, at photoinjected carrier density higher than 10^{18} cm^{-3}, the effective mass of charge carriers has been reported to increase due to the band-filling effect[7]. Since the THz signal $-\Delta E/E(t)$ is proportional to the photoconductivity σ which is further related to the carrier density $N(t)$ and charge mobility $\mu(N) = e \cdot \tau(N)/m^*(N)$ (see Manuscript), the time-resolved THz signal can be expressed as following:

$$-\Delta E/E\,(t) = c \cdot N(t) \cdot e \cdot \mu(N) \tag{1}$$

c is the proportionality factor which can be determined independently at the low carrier density regime where the mobility is a constant.

The Auger recombination follows the recombination rate equation:

$$dN(t)/dt = -k_3 \cdot N^3 \tag{2}$$

k_3 is the Auger recombination constant. Solving the differential equation, we obtain the time-dependent expression of $N(t)$.

For the carrier density-dependent effective mass $m^*(N)$, we take the calculation of Whalley et al.[7]. Putting $N(t)$ and $m^*(N)$ into Equation 1, and then we fit it globally to the fluence-dependent OPTP dynamics in **Fig. S4a**. As we can see, the fitting is adequently fine, indicating the capture of the decay essence. From the fitting, we extract the carrier density-dependent mobility and scattering time. In **Fig. S4c**, the carrier density-dependent scattering time can be well fitted by empirical Caughey-Thomas relation[6].

We conclude that the fast decay can be well described by Auger recombination with varied mobility. The signal variation with increasing charge carrier density is predominantly due to a reduction in the scattering time (factor of 5), in addition to a small contribution from an increase in effective mass (factor of 1.2).

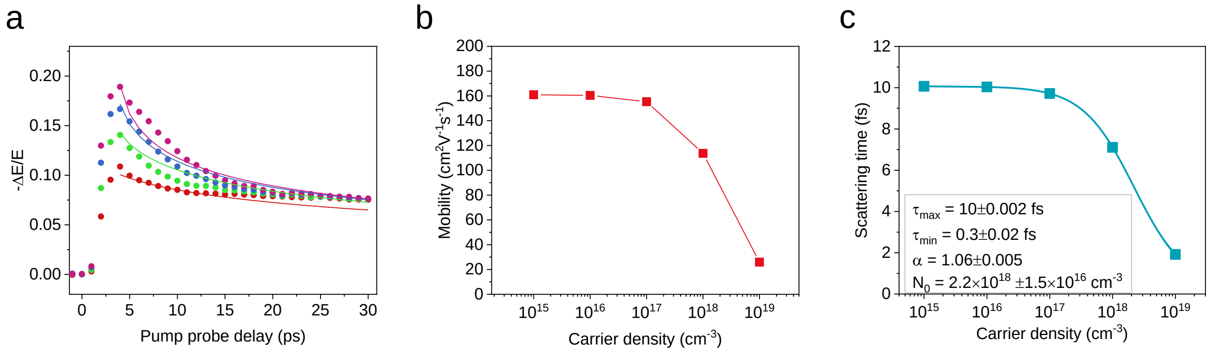

Fig. S4. a, The fluence-dependent OPTP dynamics for $MAPbI_3$ at 78K. The dots are the experimental data; the solid lines are the fitting as discussed above. **b,** The extracted charge mobility and **c,** scattering time from the fitting. The solid line in c is the fitting by Caughey-Thomas relation.

Section 5: The extraction of critical density by THz-TDS

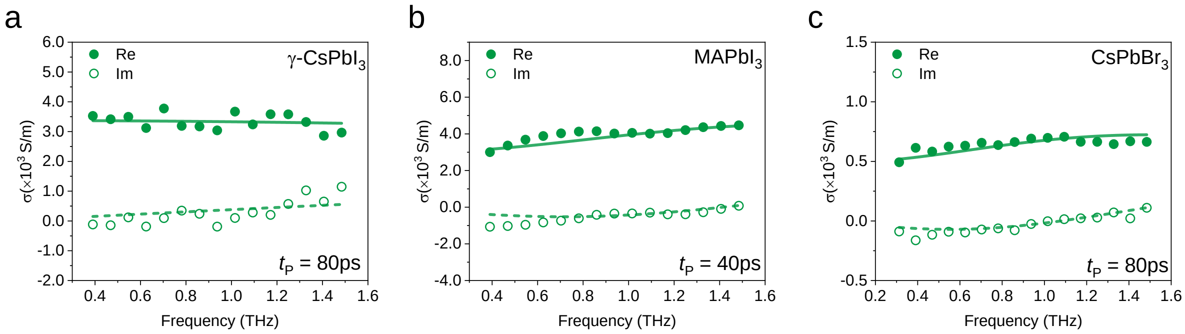

Fig. S5. The frequency-resolved complex photoconductivity spectra measured 80 ps after photoexcitations at 78 K for **a,** γ-$CsPbI_3$ at initially photoinjected carrier density of 9.35×10^{17} cm^{-3}, **b,** $MAPbI_3$ at 5×10^{18} cm^{-3}, **c,** $CsPbBr_3$ at 1.35×10^{18} cm^{-3}. The solid and dashed lines are the Drude model fit for γ-$CsPbI_3$ and Drude-Smith model fit for $MAPbI_3$ and $CsPbBr_3$.

Table S2. The extracted parameters from the above fitting

Sample	Scattering time (fs)	Carrier density (10^{17} cm^{-3})	Parameter *c*
γ-$CsPbI_3$	18 ± 4	6.62 ± 1.35	0
$MAPbI_3$	64 ± 3	5.82 ± 0.31	-0.66 ± 0.02
$CsPbBr_3$	80 ± 5	6.14 ± 0.42	-0.65 ± 0.02

Section 6: Extracted critical densities at different pump wavelength

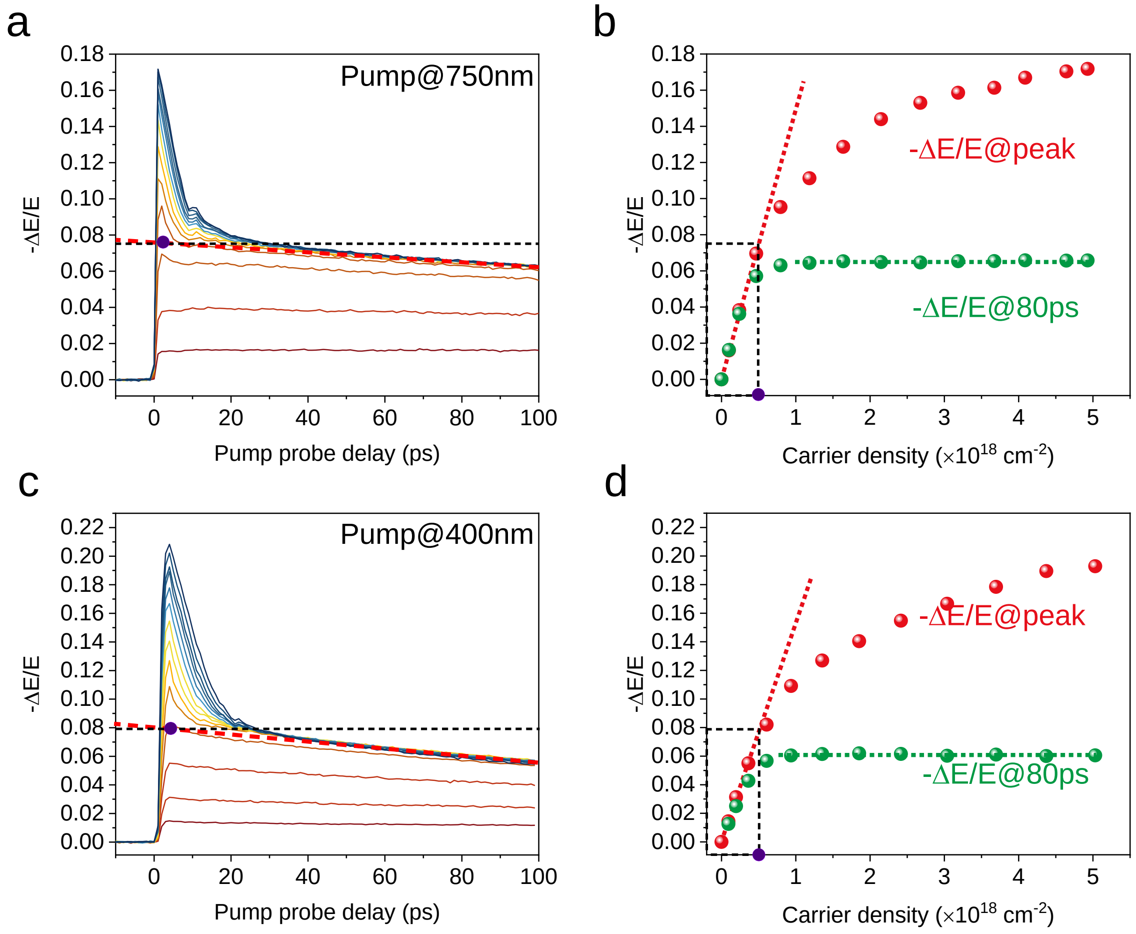

Fig. S6. The fluence-dependent OPTP dynamics and the extraction of critical density for $MAPbI_3$ at 78K at pump wavelengths of **a, b,** 750 nm and **c, d,** 400 nm. The extracted critical density for both wavelengths is around 5×10^{17} cm^{-3}, indicating the independence of the critical density on the pump wavelength.

Section 7: Mott polaron density calculation based on Feynman polaron model

Table S3. The material parameters utilized for calculating the Mott polaron density based on Feynman polaron model

Sample	ε_0	ε_∞	υ_{LO} (THz)	m* (m_0)
γ-$CsPbI_3$ [8]	18.10	6.10	2.57	0.10 [9]
$MAPbI_3$ [10]	33.50	5.00	3.38	0.10
$CsPbBr_3$ [11]	29.37	4.30	4.07	0.17 [12]

Here, ε_0 and ε_∞ are the static and optical dielectric functions in units of vacuum permittivity, υ_{LO} is the effective optical phonon frequency (by the Hellwarth et al. 'B' temperature-independent

scheme[13]), m* is the effective mass in units of rest mass m_0. These parameters specify the Fröhlich Hamiltonian. This Hamiltonian is then used in the POLARONMOBILITY.JL codes, which numerically solve (with finite temperature) the Feynman variational polaron approach. We use the Osaka extension to the Fröhlich Hamiltonian, which contains phonon entropy terms, making it a finite temperature free-energy theory. The resulting v and w variational parameters specify a quasiparticle model for the polaron, where an effective harmonic confining potential is set up by the electron-phonon coupling with the phonons. Solving for the Gaussian wavefunction in this confining potential gives us the Schultz polaron radius, from which we calculate the volume and, thereby, the polaron density.

Additionally, we investigated the non-parabolicity of effective mass with band filling, as a potential alternative hypothesis for explaining the density-dependent data. For this, we undertook a consistent set of calculations in the VASP plane-wave electronic structure codes. The band structures were calculated using the screened hybrid functional HSE06, including spin-orbit coupling (SOC). The energy cutoff for the simulations was 800 eV and the k-space sampling was a Γ centered grid of 4×4×4. After obtaining the band structure using DFT methods, the Python package 'effmass' was used to determine the electron-effective mass. For the curvature/conventional electron effective mass, the parabolic approximation to the band structure was obtained using three-point-finite differences. To calculate the optical effective mass for a range of conduction electron concentrations, the Fermi-Dirac weighted dispersion over all bands was integrated along the reciprocal space using the Kane dispersion. An average of the effective mass over the directions (001), (011) and (111) was performed.

The key result is that the three material systems consistently have the same density-dependence of effective mass as reported by Whalley et al.[7] for MAPI, justifying our use of these parameters across the three material systems.

Section 8: The extraction of critical density at different temperatures

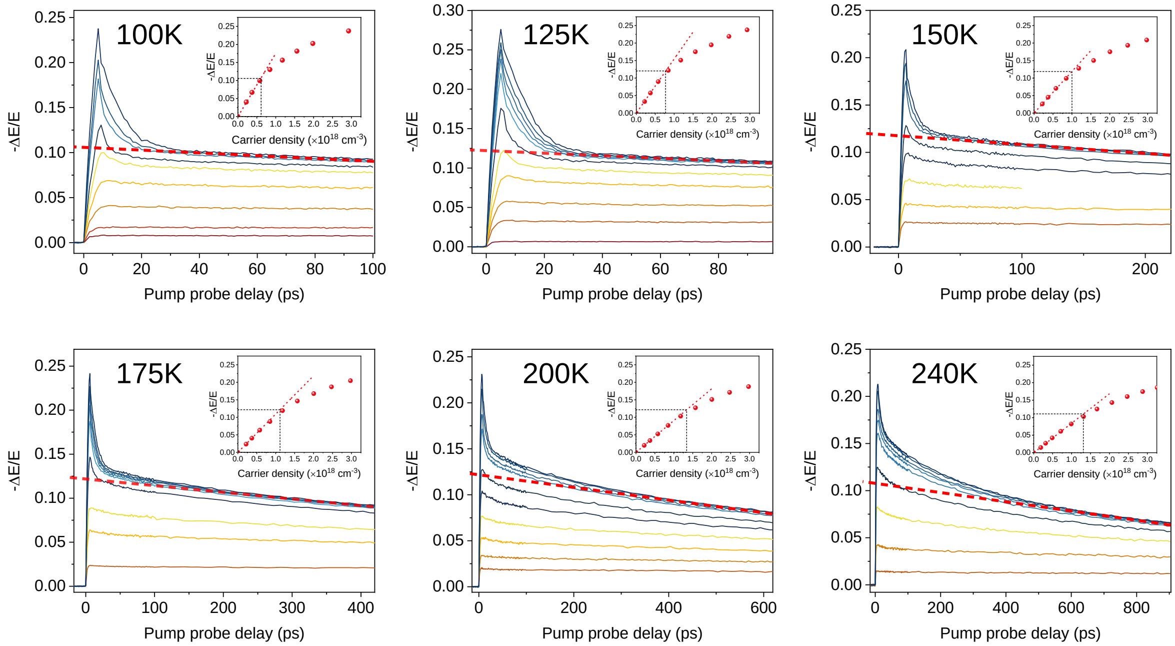

Fig. S7. The fluence-dependent OPTP dynamics and the extraction of critical density at different temperatures for γ-$CsPbI_3$.

Section 9: The photon-to-carrier conversion ratio at different temperature

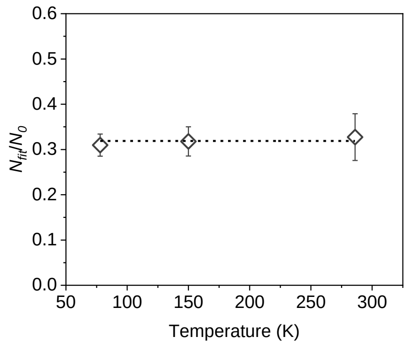

Fig. S8. The ratio of extracted carrier density N_{Fit} from Drude fitting to the incident photon density N_0 at different temperatures in black γ-$CsPbI_3$ with pump wavelength of 400 nm and incident photon density of 1.33×10^{13} cm^{-2}.

Section 10: Density-dependent effective mass and charge mobility in γ-$CsPbI_3$

The effective mass is expected to decrease when the carrier density exceeds the Mott density. This is because beyond the Mott transition, charge carriers are expected to be more band-like charge carriers (e.g., behave more like bare charges). We can estimate the upper limit of the effective mass reduction following Feynman's polaron theory. The polaron effective mass m_p^* can be well approximated as:[14]

$$m_p^* = m_b^*(1 + \frac{\alpha}{6} + \frac{\alpha^2}{40} + \cdots)$$

Here, m_b^* is the bare band mass, α is the dimensionless Fröhlich electron-phonon coupling constant. In lead halide perovskites, α has been widely reported to be around 2[1,10]. In our previous report[1], we have calculated α for black phase $CsPbI_3$ to be 1.23, suggesting a 24% enhancement of the polaron effective mass compared to the bare band mass. On the other hand, the scattering time of the charge carriers reduces more than 50% from >40 fs to <20 fs going from below to above the Mott transition (as shown in Fig. 4a in the main text). The mobility is determined by both the effective mass and scattering time following $\mu = \frac{e \cdot \tau}{m^*}$. The bare band mass in black phase $CsPbI_3$ has been reported to be $0.1m_0$ as shown in Table S3, and the polaron effective mass is calculated to be 0.124 m_0 according to the above equation. As such, we find that the charge mobility in black phase $CsPbI_3$ at 78K actually decreases from ~568 $cm^2V^{-1}s^{-1}$ at polaron densities well below the Mott density, to 352 $cm^2V^{-1}s^{-1}$ above Mott density, *despite* the reduction in effective mass.

References

1 Zhang, H., Debroye, E., Steele, J. A., Roeffaers, M. B. J., Hofkens, J., Wang, H. I. & Bonn, M. Highly mobile large polarons in black phase $CsPbI_3$. *ACS Energy Lett.* **6**, 568-573 (2021).

2 Yin, Y., Fu, S., Zhou, S., Song, Y., Li, L., Zhang, M., Wang, J., Mariyappan, P., Alshehri, S. M., Ahamad, T. & Yamauchi, Y. Efficient and stable ideal bandgap perovskite solar cell achieved by a small amount of tin substituted methylammonium lead iodide. *Electron. Mater. Lett.* **16**, 224-230 (2020).

3 Leguy, A. M., Azarhoosh, P., Alonso, M. I., Campoy-Quiles, M., Weber, O. J., Yao, J., Bryant, D., Weller, M. T., Nelson, J., Walsh, A., van Schilfgaarde, M. & Barnes, P. R. Experimental and theoretical optical properties of methylammonium lead halide perovskites. *Nanoscale* **8**, 6317-6327 (2016).

4 Ulbricht, R., Hendry, E., Shan, J., Heinz, T. F. & Bonn, M. Carrier dynamics in semiconductors studied with time-resolved terahertz spectroscopy. *Rev. Mod. Phys.* **83**, 543-586 (2011).

5 Hendry, E., Koeberg, M., Pijpers, J. & Bonn, M. Reduction of carrier mobility in semiconductors caused by charge-charge interactions. *Phys. Rev. B* **75**, 233202 (2007).

6 Mics, Z., D'Angio, A., Jensen, S. A., Bonn, M. & Turchinovich, D. Density-dependent electron scattering in photoexcited GaAs in strongly diffusive regime. *Appl. Phys. Lett.* **102**, 231120 (2013).

7 Whalley, L. D., Frost, J. M., Morgan, B. J. & Walsh, A. Impact of nonparabolic electronic band structure on the optical and transport properties of photovoltaic materials. *Phys. Rev. B* **99**, 085207 (2019).

8 Frost, J. M. Calculating polaron mobility in halide perovskites. *Phys. Rev. B* **96**, 195202 (2017).

9 Kang, Y. & Han, S. Intrinsic carrier mobility of cesium lead halide perovskites. *Phys. Rev.*

Appl. **10**, 044013 (2018).
10 Sendner, M., Nayak, P. K., Egger, D. A., Beck, S., Müller, C., Epding, B., Kowalsky, W., Kronik, L., Snaith, H. J., Pucci, A. & Lovrinčić, R. Optical phonons in methylammonium lead halide perovskites and implications for charge transport. *Mater. Horiz.* **3**, 613-620 (2016).
11 Miyata, K., Meggiolaro, D., Trinh, M. T., Joshi, P. P., Mosconi, E., Jones, S. C., De Angelis, F. & Zhu, X.-Y. Large polarons in lead halide perovskites. *Sci. Adv.* **3**, e1701217 (2017).
12 Puppin, M., Polishchuk, S., Colonna, N., Crepaldi, A., Dirin, D. N., Nazarenko, O., De Gennaro, R., Gatti, G., Roth, S., Barillot, T., Poletto, L., Xian, R. P., Rettig, L., Wolf, M., Ernstorfer, R., Kovalenko, M. V., Marzari, N., Grioni, M. & Chergui, M. Evidence of large polarons in photoemission band mapping of the perovskite semiconductor $CsPbBr_3$. *Phys. Rev. Lett.* **124**, 206402 (2020).
13 Hellwarth, R. W. & Biaggio, I. Mobility of an electron in a multimode polar lattice. *Phys. Rev. B* **60**, 299 (1999).
14 Feynman, R. P. Slow Electrons in a Polar Crystal. *Phys. Rev.* **97**, 660 (1955).
